# Gene expression profiles in thyroid carcinomas

**DOI:** 10.1054/bjoc.2000.1483

**Published:** 2000-11-22

**Authors:** T Takano, Y Hasegawa, F Matsuzuka, A Miyauchi, H Yoshida, T Higashiyama, K Kuma, N Amino

**Affiliations:** Department of Laboratory Medicine, Surgical Oncology, Osaka University Medical School, D2 2-2 Yamadaoka, Suita, Osaka, 565-0871; Kuma Hospital, 8-2-35, Simoyamatedori, Chuo-ku, Kobe, Hyogo, 650-0011, Japan

**Keywords:** gene expression, SAGE, gene therapy, anaplastic carcinoma, follicular carcinoma, molecular-based diagnosis

## Abstract

The gene expression profiles of human thyroid carcinomas were analysed by serial analysis of gene expression (SAGE) which allows quantitative and simultaneous analysis of a large number of transcripts. More than 29 000 transcripts derived from a normal thyroid tissue and four thyroid tumours were analysed. While extensive similarity was noted between the expression profiles of the normal thyroid tissue and three differentiated thyroid tumours, many transcripts, such as osteonectin, a-tubulin, glyceraldehyde-3-phosphate dehydrogenase, glutathione peroxidase, and thyroglobulin, were expressed at extremely different levels in differentiated and undifferentiated carcinomas. These data provide new information that might be used to identify genes useful for the diagnosis and treatment of thyroid carcinomas. © 2000 Cancer Research Campaign http://www.bjcancer.com


*British Journal of Cancer* (2000) **83**(11), 1495–1502
© 2000 Cancer Research Campaign
doi: 10.1054/ bjoc.2000.1483, available online at http://www.idealibrary.com on IDEAL®
http://www.bjcancer.com

### Gene expression profiles in thyroid carcinomas

**T Takano[1], Y Hasegawa[1], F Matsuzuka[3], A Miyauchi[3], H Yoshida[3], T Higashiyama[2], K Kuma[3] and N Amino[1]**

[1]Department of Laboratory Medicine, and [2] Surgical Oncology, Osaka University Medical School, D2, 2-2 Yamadaoka, Suita, Osaka 565-0871, [3]Kuma Hospital, 8-2-35, Simoyamatedori, Chuo-ku, Kobe, Hyogo 650-0011, Japan

Summary The gene expression profiles of human thyroid carcinomas were analysed by serial analysis of gene expression (SAGE) which allows quantitative and simultaneous analysis of a large number of transcripts. More than 29 000 transcripts derived from a normal thyroid tissue and four thyroid tumours were analysed. While extensive similarity was noted between the expression profiles of the normal thyroid tissue and three differentiated thyroid tumours, many transcripts, such as osteonectin, a-tubulin, glyceraldehyde-3-phosphate dehydrogenase, glutathione peroxidase, and thyroglobulin, were expressed at extremely different levels in differentiated and undifferentiated carcinomas. These data provide new information that might be used to identify genes useful for the diagnosis and treatment of thyroid carcinomas. © 2000 Cancer Research Campaign http://www.bjcancer.com

**Keywords:** gene expression; SAGE; gene therapy; anaplastic carcinoma; follicular carcinoma; molecular-based diagnosis

Recent advances in molecular technology suggest the potential for more efficient and effective molecular-based diagnoses and therapies. Many studies, such as those concerning *p53, RAS, RET,* and thyrotropin receptor, have improved our understanding of thyroid carcinogenesis (Farid, 1996). However, more intensive studies to further clarify the molecular mechanism of carcinogenesis are necessary before we select the molecular targets for these technologies.

In the thyroid, as in other organs, genes that are found to be differentially expressed between normal thyroid tissue and thyroid carcinomas can be used as targets for molecular-based diagnosis and therapy (Chiappetta et al, 1998; Takano et al, 1998, 1999). Recent developments in technologies aimed at identifying differentially expressed genes, such as differential hybridization and differential display, have identified some genes the expression of which is restricted to thyroid carcinomas (Gonsky et al, 1997; Takano et al, 1997; de Nigris et al, 1998). However, the data made available by these methods are still insufficient for a comprehensive evaluation of all genes involved in carcinogenesis.

By relying on 14–15 base cDNA sequences for gene identification, serial analysis of gene expression (SAGE) can generate a quantitative transcript profile easily, a task currently not possible using alternative transcript imaging technologies (Velculescu et al, 1995), and is less laborious than the body mapping method which can generate similar profiles (Matsubara and Okubo, 1993). Since its introduction in 1995, SAGE has been used to analyse cDNA libraries derived from several carcinomas and its reliability has been established (Zhang et al, 1997; Hibi et al, 1998). We describe here the use of SAGE to provide gene expression profiles in normal thyroid and thyroid tumours, a technique that may lead to an enhanced understanding of thyroid cell function and carcinogenesis.

*Received 3 May 2000*
*Revised 26 July 2000*
*Accepted 10 August 2000*

*Correspondence to:* Toru Takano

### MATERIALS AND METHODS

### Materials

Tissue samples for SAGE were obtained surgically from a normal thyroid tissue adjacent to a follicular adenoma in a 43-year-old female, a follicular adenoma in a 43-year-old female, a papillary carcinoma in a 32-year-old female, a widely invasive follicular carcinoma in a 35-year-old female, and an anaplastic carcinoma in a 77-year-old female. Tissue samples from three normal thyroids, follicular adenomas, papillary carcinomas, follicular carcinomas and anaplastic carcinomas were also collected for reverse transcription-polymerase chain reaction (RT-PCR) analysis. Thyroid tumours were classified histopathologically according to the WHO histological classification of thyroid tumours (Hedinger et al, 1989). Total cellular RNA was extracted according to the method of Chomczynski and Sacchi (Chomczynski and Sacchi, 1987) and poly A RNA was purified with oligotex-dT30 (Takara, Shiga, Japan) according to the manufacturer’s protocol.

### SAGE protocol

The SAGE method was performed as described previously with some modifications. 3 μg of poly A RNA was converted to double-stranded cDNA with a BRL synthesis kit (Gibco BRL, Tokyo, Japan) according to the manufacturer’s protocol except for the inclusion of primer biotin-5′-$T_{18}$-3′. The cDNA was cleaved with Nla III (anchoring enzyme) (Daiichi-Kagaku, Tokyo, Japan). After capture of the 3′ cDNA fragments on streptavidin-coated magnetic beads (Dynal, Tokyo, Japan), the bound cDNA was divided into two pools, and one of the following linkers containing a recognition site for Bsm FI (Daiichi-Kagaku) was ligated to each pool:

linker 1, 5′-TTTGGATTTGCTGGTGCAGTACAACTAG-GCTTAATAGGGACATG-3′, 5′-TCCCTATTAAGCC-TAGTTGTAXTGCACCAGCAAATCC (amino modification C7)-3′;
linker 2, 5′-TTTCTGCTCGAATTCAAGCTTCTAACGATG-TACGGGGACATG-3′, 5′-TCCCCGTACATCGTTA-GAAGCTTGAATTCGAGCAG (amino modification C7)-3′.

Since Bsm FI (tagging enzyme) cleaves 14 bp away from its recognition site, and the Nla III site overlaps the Bsm FI site by 1 bp, a 15 bp SAGE tag was released with Bsm FI, SAGE tag overhangs were filled in with Klenow (Takara), and tags from the two pools were combined and ligated to each other. The ligation product was amplified by 15 cycles of PCR using 5′-GGATTTGCTGGTGCAGTACA-3′ and 5′-CTGCTCGAAT-TCAAGCTTCT-3′ as primers. All the linkers and primers were obtained from Gibco BRL. The PCR products were analysed by polyacrylamide gel electrophoresis (PAGE), and the PCR product containing two tags ligated tail to tail (ditag) was excised. The PCR product was re-amplified by 20 cycles of PCR using the same primers, purified by PAGE, then cleaved with Nla III. The band containing the ditags was excised and self-ligated, then cleaved with Sph I (Takara). The concatenated products were separated by gel filtration using a Sephadex 400R (Amersham Pharmacia, Tokyo, Japan), then cloned into the Sph I site of pGEM-5Zf (+) (Promega, Tokyo, Japan). These procedures produced about 500 white colonies per reaction. Colonies were screened for inserts by PCR using primers which sequences located outside the cloning site. Colonies containing inserts of about 400 bp in length were selected for the further analysis. Plasmids from selected clones were purified by an automatic plasmid isolation system PI-100Σ (Kurabo, Osaka, Japan) then sequenced with Taq FS Dye Primer kits (PE Biosystems, Tokyo, Japan) and analysed using a 373 ABI automated sequencer (PE Biosystems), following the manufacturer's protocol. Sequence files were analysed by the SAGE software and the tag sequences were analysed by the BLAST program of the DNA Data Bank of Japan (Mishima, Sizuoka, Japan). The occurrence rates of tag sequences were calculated by dividing the number of occurrences of a particular tag sequence by the total tag count.

### Semi-quantitative RT-PCR analysis

Semi-quantitative RT-PCR analyses of 4 representative mRNA sequences were performed as previously described (Takano et al, 1997). The sequence of the 5′ primers are 5′-GGATTTGCTGGT-GCAGTACA-3′ (base 1511–1530) (Swaroop et al, 1988) for

**Table 1** SAGE analysis of a normal thyroid and a follicular adenoma

**Normal thyroid**
**Total no. of tags = 5411, no. of unique tags = 623**

| Count | Sequence | Definition |
|---|---|---|
| 64 | CCACTGCACT | EST A1081056 |
| 63 | CGGTGAAAAA | thyroglobulin |
| 55 | ACTTTTTCAA | mitochondrial cytochrome oxidase subunit 1 |
| 50 | GTGAAACCCC(G) | 1. Alu transcript<br>2. obese protein<br>3. platelet-activating factor acetylhydrolase 2 |
| 49 | CCTGTAATCCC | 5'-nucleotidase |
| 48 | GTGAAACCCC(A) | 1. granulocyte-macrophage colony-stimulating factor receptor alpha-subunit soluble isoform 2<br>2. myelin/oligodendrocyte glycoprotein-25.1kD<br>3. fibroblast growth factor receptor |
| 33 | GTGAAACCCT | putative serine-thereonine protein kinase |
| 30 | TGTGTTGAGA | elongation factor 1-alpha |
| 29 | CACCTAATTG | mitochondrial ATP synthase 6 |
| 28 | AACCCGGGAG | 1. transmembrane receptor protein<br>2. primary Alu transcript |
| 28 | CCCATCGTCC | mitochondrial cytochrome oxidase subunit 2 |
| 26 | AGGGAGGGGC | glutathione peroxidase |
| 25 | TCAAGCCATC | EST AI563994 |
| 25 | TTCATACACC | mitochondrial NADH dehydrogenase 4 |
| 23 | CTCCACCCGA | secretory protein |
| 23 | TACATAATTA | trophoblast STAT |
| 20 | AGCCCTACAA | mitochondrial NADH dehydrogenase 3 |
| 20 | GCGAAACCCC | EST N71314 |
| 19 | AACCTGGGAG | DNA fragmentation factor-45 |
| 19 | AGCTCTCCCT | putative ribosomal protein L23 |
| 18 | CTAAGACTTC | EST C04521 |
| 18 | TTGGCTTGCT | EST AA515148 |
| 15 | ACCCTTGGCC | mitochondrial NADH dehydrogenase 1 |
| 15 | AGGTCAGGAG | human carcinoma cell-derived Alu RNA transcript, clone CD139 |
| 15 | CAAGCATCCC | EST A1557493 |
| 15 | CGCCGCCGGC | ribosomal protein L35 |

**Follicular adenoma**
**Total no. of tags = 5030, no. of unique tags = 569**

| Count | Sequence | Definition |
|---|---|---|
| 144 | CGGTGAAAAA | thyroglobulin |
| 56 | CCTGTAATCC | 5′-nucleotidase |
| 54 | ACTTTTTCAA | mitochondrial cytochrome oxidase subunit 1 |
| 54 | CCACTGCACT | EST AI081056 |
| 47 | CGGTGAAGCA | no match |
| 38 | GTGAAACCCT | putative serine-threonine protein kinase |
| 34 | TGTGTTGAGA | elongation factor 1-alpha |
| 33 | GTGAAACCCC(G) | 1. Alu transcript<br>2. obese protein<br>3. platelet-activating factor acetylhydrolase 2 |
| 29 | GTGAAACCCC(A) | 1. granulocyte-macrophage colony-stimulating factor receptor alpha-subunit soluble isoform 2<br>2. myelin/oligodendrocyte glycoprotein-25.1kD<br>3. fibroblast growth factor receptor |
| 23 | TAGGTTGTCT | translationally controlled tumor protein |
| 20 | CACCTAATTG | mitochondrial ATP synthase 6 |
| 19 | AGCTCTCCCT | putative ribosomal protein L23 |
| 19 | GGCAAGCCCC | Csa-19 |
| 19 | TTCATACACC | mitochondrial NADH dehydrogenase 4 |
| 19 | TTGGTCCTCT | ribosomal protein L41 |
| 18 | CCCATCGTCC | mitochondrial cytochrome oxidase subunit 2 |
| 17 | AACCCGGGAG | 1. transmebrane receptor protein<br>2. primary Alu transcript |
| 17 | AGCCCTACAA | mitochondrial NADH dehydrogenase 3 |
| 16 | CCTCAGGATA | mitochondrial NADH dehydrogenase 6 |
| 16 | GCCGAGGAAG | ribosomal protein S12 |
| 15 | CACAAACGGT | 1. metallopanstimulin<br>2. ribosomal protein S27 |
| 14 | CCTGTAGTCC | EST R10346 |
| 14 | TTGGCCAGGC | 1. aggrecanase-1<br>2. interferon-inducible RNA-dependent protein kinase<br>3. glucose-6-phosphatase |
| 13 | CCAGAACAGA | 1. ribosomal protein L30<br>2. thymidylate kinase |
| 14 | GACGACACGA | ribosomal protein S28 |

 *© 2000 Cancer Research Campaign*

**Table 2** SAGE analysis of papillary, follicular and anaplastic carcinomas

**Papillary carcinoma**
**Total no. of tags = 6435, no. of unique tags = 662**

| Count | Sequence | Definition |
|---|---|---|
| 159 | CCCATCGTCC | mitochondrial cytochrome oxidase subunit 2 |
| 146 | CACCTAATTG | mitochondrial ATP synthase 6 |
| 122 | ACCCTTGGCC | mitochondrial NADH dehydrogenase 1 |
| 93 | TGATTTCACT | mitochondrial cytochrome c oxidase subunit 3 |
| 84 | TTGGGGTTTC | ferritin H chain |
| 79 | GTGAAACCCC(G) | 1. Alu transcript |
| | | 2. obese protein |
| | | 3. platelet-activating factor acetylhydrolase 2 |
| 77 | ACTAACACCC | mitochondrial NADH dehydrogenase 2 |
| 65 | TTCATACACC | mitochondrial NADH dehydrogenase 4 |
| 64 | TTGGTCCTCT | ribosomal protein L41 |
| 56 | ACTTTTTCAA | mitochondrial cytochrome oxidase subunit 1 |
| 56 | CAAGCATCCC | EST AI557493 |
| 56 | CACTACTCAC | mitochondrial cytochrome b |
| 51 | AGCCCTACAA | mitochondrial NADH dehydrogenase 3 |
| 46 | TGTGTTGAGA | elongation factor 1-alpha |
| 44 | CCACTGCACT | EST AI081056 |
| 44 | CCTGTAATCC | 5′-nucleotidase |
| 37 | CTAAGACTTC | EST C04521 |
| 35 | CGGTGAAAAA | thyroglobulin |
| 33 | TCGAAGCCCC | EST AA533220 |
| 31 | GCCGAGGAAG | ribosomal protein S12 |
| 30 | TGGGTGAGCC | cathepsin B |
| 28 | AAGACAGTGG | ribosomal protein L37a |
| 27 | AGCACCTCCA | elongation factor 2 |
| 26 | GGACCACTGA | ribosomal protein L3 |
| 23 | GCAGCCATCC | ribosomal protein L28 |

**Follicular carcinoma**
**Total no. of tags = 5275, no. of unique tags = 630**

| Count | Sequence | Definition |
|---|---|---|
| 188 | CGGTGAAAAA | thyroglobulin |
| 55 | CCTGTAATCC | 5′-nucleotidase |
| 51 | CCCATCGTCC | mitochondrial cytochrome oxidase subunit 2 |
| 45 | AGCCCTACAA | mitochondrial NADH dehydrogenase 3 |
| 44 | CACCTAATTG | mitochondrial ATP synthase 6 |
| 38 | CCACTGCACT | EST AI081056 |
| 34 | GTGAAACCCC(G) | 1. Alu transcript |
| | | 2. obese protein |
| | | 3. platelet-activating factor acetylhydrolase 2 |
| 30 | CGGTGAAGCA | no match |
| 29 | AGGGAGGGGC | glutathione peroxidase |
| 29 | TTGGTCCTCT | ribosomal protein L41 |
| 28 | AGGGCTTCCA | Wilm’s tumor-related protein |
| 26 | ACCCTTGGCC | mitochondrial NADH dehydrogenase 1 |
| 26 | TGTGACGCCG | no match |
| 23 | ACTAACACCC | mitochondrial NADH dehydrogenase 2 |
| 23 | TGGGTGAGCC | cathepsin B |
| 21 | CACAAACGGT | 1. metallopanstimulin |
| | | 2. ribosomal protein S27 |
| 20 | AAGACAGTGG | ribosomal protein L37a |
| 20 | CGCCGCCGGC | ribosomal protein L35 |
| 20 | GTGAAACCCT | putative serine-threonine protein kinase |
| 19 | TGTGTTGAGA | elongation factor 1-alpha |
| 19 | TTCATACACC | mitochondrial NADH dehydrogenase 4 |
| 18 | GTGAAACCCC(A) | 1. granulocyte-macrophage colony-stimulating factor receptor alpha-subunit soluble isoform 2 |
| | | 2. myelin/oligodendrocyte glycoprotein-25.1kD |
| | | 3. fibroblast growth factor receptor transmembrane form |
| 18 | AACCCGGGAG | 1. transmebrane receptor protein |
| | | 2. primary Alu transcript |
| 18 | CCATTGCACT | EST T07339 |
| 18 | GCAGCCATCC | ribosomal protein L28 |

**Anaplastic carcinoma**
**Total no. of tags = 7124, no. of unique tags = 849**

| Count | Sequence | Definition |
|---|---|---|
| 87 | ACTTTTTCAA | mitochondrial cytochrome oxidase subunit 1 |
| 64 | CCCATCGTCC | mitochondrial cytochrome oxidase subunit 2 |
| 63 | GTTGTGGTTA | beta 2-microglobulin |
| 60 | ATGTGAAGAG | *SPARC*/osteonectin |
| 59 | TTCATACACC | mitochondrial NADH dehydrogenase 4 |
| 55 | TGGAAATGAC | alpha-1 collagen (polymorphic transcript) |
| 51 | GTTCACATTA | HLA-DR antigens associated invariant chain |
| 41 | AGCCCTACAA | mitochondrial NADH dehydrogenase 3 |
| 41 | CACCTCCTAT | no match |
| 40 | GGATTTGGCC | acidic ribosomal phosphoprotein P2 |
| 40 | GTGTGTTTGT | transforming growth factor-beta induced gene product |
| 37 | ACCAAAAACC | alpha-1 collagen type 1 |
| 37 | CCAGAACAGA | 1. ribosomal protein L30 |
| | | 2. thymidylate kinase |
| 34 | CCTAGCTGGA | T-cell cyclophilin |
| 32 | GAGGGAGTTT | ribosomal protein L27a |
| 29 | TACCATCAAT | glyceraldehyde-3-phosphate dehydrogenase |
| 28 | CCACTGCACT | EST AI081056 |
| 28 | TTGGGGTTTC | ferritin H chain |
| 27 | AGGGCTTCCA | Wilm’s tumour-related protein |
| 27 | CACCTAATTG | mitochondrial ATP synthase 6 |
| 27 | CCCTGGGTTC | ferritin light subunit |
| 27 | TAGGTTGTCT | translationally controlled tumour protein |
| 27 | TTGGTCCTCT | ribosomal protein L |
| 26 | AAGGTGGAGG | ribosomal protein L18a |
| 26 | AGAAAAAAAA | no match |

© 2000 Cancer Research Campaign

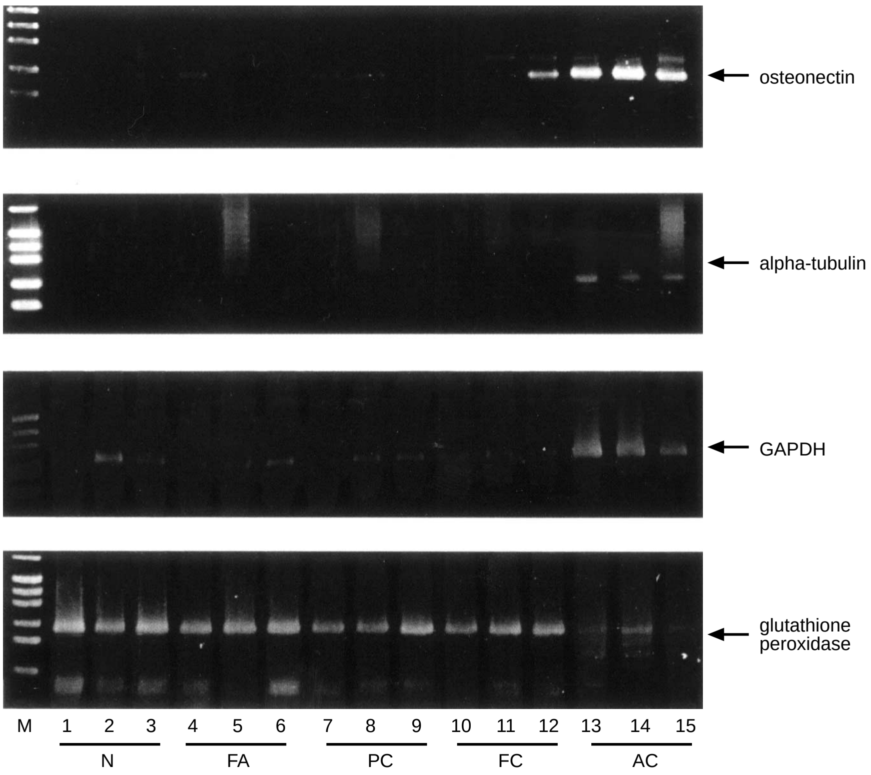

**Figure 1** Semi-quantitative RT-PCR analysis of osteonectin, α-tubulin, GAPDH, and glutathione peroxidase mRNAs. Tissue samples from three normal thyroids (N), follicular adenomas (FA), papillary carcinomas (PC), follicular carcinomas (FC) and anaplastic carcinomas (AC) were subjected to RT-PCR analysis. PCR products were run on a 1.5% agarose gel, then the gel was stained with SYBR Green 1 (Takara). Arrows indicate the expected positions of the PCR products. M: PHY maker (Takara)

osteonectin, 5′-GGATTTGCTGGTGCAGTACA-3′ (base 1021–1040) for α-tubulin (Cowan et al, 1983), 5′-CCAAGGTCATCCAT-GACAAC (base 557–576) for glyceraldehydes-3-phosphate dehydrogenase (GAPDH) (Arcari et al, 1984), and 5′-ACGTGTC-CTACCTATGTGTC-3′ (base 981–1000) for glutathione peroxidase (Takahashi et al, 1990). A poly A-anchor primer DDR (5′-ATGCGAATTCGTTTTTTTTTTTTTTTTTTT-3′) was used for the 3′ primer. RT was performed using 1 μg of total RNA in an RT mixture containing 40 mM Tris-HCl (pH 8.3), 75 mM KCl, 10 mM DTT, 3 mM $MgCl_2$, 0.5 mM dNTPs, 200 U Moloney murine leukaemia virus reverse transcriptase (Gibco BRL), 2 U/μl RNase inhibitor (Takara), and 2.5 μM oligodeoxythymidylic acid (Gibco BRL) in a total volume of 20 μl at 37˚C for 60 min. For PCR, each reaction mixture consist of 1 μl of cDNA, 0.5 μM each primer, 2 μl of 10 × Ex Taq buffer (Takara), 1.6 μl of 2 mM dNTP mix (PE Biosystems) 0.5 U of Ex Taq polymerase (Takara), and nuclease-free water to a final volume of 20 μl. The reaction mixture was subjected to 25 cycles of denaturation (94˚C; 1 min), annealing (55˚C; 1 min), andextension (72˚C; 1 min). After PCR amplification, 5 μl of reaction mixture was run on 1.5% agarose gel. The gel was stained with SYBR Green I (Takara), then analysed with a Fluor Imager (Molecular Dynamics, Sunnyvale, CA).

### RESULTS

SAGE libraries were constructed from mRNAs isolated from a normal thyroid tissue sample and four thyroid tumours. In total, 29 275 tags were sequenced, representing about 600 unique tags in each tissue (Tables 1 and 2). The majority of the highly expressed sequences in each tissue code mitochondorial and ribosomal proteins. The tag sequence of thyroglobulin mRNA was highly expressed in the normal thyroid and the 3 differentiated thyroid tumours but not in the anaplastic carcinoma. In the 2 differentiated carcinomas, high expression levels of the tag sequence of cathepsin B were observed. In the anaplastic carcinoma, most of the highly occurring tag sequences were derived from housekeeping genes in addition to mitochondorial and ribosomal sequences. Some sequences that were only seldom observed in the differentiated carcinomas, such as those of osteonectin and collagen genes, were also highly expressed.

To generate a profile of the relative gene expression patterns in each tumour, the occurrences of each tag identified in the tumour library were compared with those observed in the libraries of the other tumours or of the normal thyroid. Representative sequences are listed in Tables 3 and 4. The tag sequences that code mitochondorial and ribosomal proteins were excluded from the lists. A small number of tag sequences showed extreme differences in the expression levels among the normal thyroid and differentiated tumours. In contrast, among the 97 tag sequences which occurred 10 times or more, 29 (29.8%) and 27 (27.8%) sequences occurred at rates 10-fold or more than those in papillary and follicular carcinomas, respectively, which indicates that the expression profile of the anaplastic carcinoma is much different from those of the differentiated carcinomas.

Expression levels of some genes whose tag sequences were differentially expressed in the anaplastic carcinoma were examined by semi-quantitative RT-PCR. Semi-quantitative RT-PCR

 *© 2000 Cancer Research Campaign*

**Table 3** List of differentially expressed genes in the normal thyroid (N), follicular adenoma (F), papillary carcinoma (PC), and follicular carcinoma (FC)

| Count N | Count F | Sequence |
|---|---|---|
| 25 | 0 | TCAAGCCATC |
| EST AI563994 | | |
| 18 | 0 | TTGGCTTGCT |
| EST AA515148 | | |
| 14 | 1 | GAAATAAAGC |
| germline immunoglobulin gamma 1 chain constant region | | |
| 14 | 0 | CCCAACGCGC |
| alpha globin | | |
| 13 | 1 | AAGGGAGCAC |
| Ig germline lambda-chain | | |
| 13 | 1 | GGATATGTGG |
| transcription factor ETR103 | | |
| *n* = 6 | | |

| Count F | Count PC | Sequence |
|---|---|---|
| 1 | 20 | GCAAGCCAAC |
| EST AA133564 | | |
| 1 | 18 | ACACAGCAAG |
| EST AA654674 | | |
| 1 | 17 | GCGACCGTCA |
| aldolase A | | |
| 0 | 13 | ACCTTGTGCC |
| L-iditol-2 dehydrogenase | | |
| 0 | 13 | GCCATCCCCT |
| mRNA from HIV-associated non-Hodgkin's lymphoma (clone h12-129) | | |
| 0 | 11 | ATGGCTGGTA |
| LLRep3 | | |
| 0 | 10 | CTGACCTGTG |
| MHC HLA-B7 class I cell surface glycoprotein heavy chain | | |
| *n* = 12 | | |

| Count F | Count FC | Sequence |
|---|---|---|
| 0 | 26 | TGTGACGCCG |
| no match | | |
| 23 | 3 | TAGGTTGTCT |
| translationally controlled tumour protein | | |
| 0 | 14 | GGAGGTGGG |
| 1. granulin | | |
| 2. epithelin 1 and 2 | | |
| 0 | 13 | GGGGAAATC |
| thymosin beta 10 | | |
| 0 | 10 | ACCAAAAACC |
| alpha-1 collagen type 1 | | |
| *n* = 5 | | |

*n*: the number of sequences occurred at rates ten-fold or more than those in the compared tissue.

© 2000 Cancer Research Campaign

**Table 4** List of differentially expressed genes in the papillary (PC), follicular (FC), and anaplastic (AC) carcinomas

| Count | | |
|---|---|---|
| **PC** | **FC** | **Sequence** |
| 0 | 26 | TGTGACGCCG |
| no match | | |
| 11 | 0 | AAAACATTCT |
| EST AA095120 | | |
| 10 | 0 | CTGACCTGTG |
| MH C HLA-B7 class I cell surface glycoprotein heavy chain | | |
| *n* = 7 | | |

| Count | | |
|---|---|---|
| **PC** | **AC** | **Sequence** |
| 4 | 60 | ATGTGAAGAG |
| *SPARC*/osteonectin | | |
| 8 | 51 | GTTCACATTA |
| HLA-DR antigens associated invariant chain | | |
| 0 | 55 | TGGAAATGAC |
| alpha-1 collagen (polymorphic transcript) | | |
| 0 | 40 | GTGTGTTTGT |
| transforming growth factor-beta induced gene product | | |
| 0 | 37 | ACCAAAAACC |
| alpha-1 collagen type 1 | | |
| 33 | 4 | TCGAAGCCCC |
| EST AA533220 | | |
| 35 | 0 | CGGTGAAAAA |
| thyroglobulin | | |
| 2 | 29 | TACCATCAAT |
| glyceraldehyde-3-phosphate dehydrogenase | | |
| 1 | 26 | TTGACACTTT |
| no match | | |
| 0 | 26 | AGAAAAAAAA |
| no match | | |
| 2 | 23 | GGGCATCTCT |
| HLA-DR alpha-chain | | |
| 2 | 21 | TGTACCTGTA |
| alpha-tubulin | | |
| 1 | 21 | CTTGTAATCC |
| EST H87461 | | |
| *n* = 45 | | |

| Count | | |
|---|---|---|
| **FC** | **AC** | **Sequence** |
| 188 | 0 | CGGTGAAAAA |
| thyroglobulin | | |
| 5 | 63 | TTGTGGTTAA |
| beta 2-microglobulin | | |
| 3 | 60 | ATGTGAAGAG |
| SPARC/osteonectin | | |
| 4 | 51 | GTTCACATTA |
| HLA-DR antigens associated invariant chain | | |
| 0 | 41 | CACCTCCTAT |
| no match | | |
| 0 | 40 | GTGTGTTTGT |
| transforming growth factor-beta induced gene product | | |
| 1 | 29 | TACCATCAAT |
| glyceraldehyde-3-phosphate dehydrogenase | | |
| 0 | 26 | AGAAAAAAAA |
| no match | | |
| 0 | 26 | TTGACACTTT |
| no match | | |
| 30 | 0 | CGGTGAAGCA |
| no match | | |
| 29 | 0 | AGGGAGGGGC |
| glutathione peroxidase | | |
| 1 | 21 | TGTACCTGTA |
| alpha-tubulin | | |
| 1 | 21 | TTGCTGACTT |
| collagen VI alpha-1 | | |
| *n* = 34 | | |

*n*: the number of sequences occurred at rates ten-fold or more than those in the compared tissue.

*© 2000 Cancer Research Campaign*

confirmed increased expression of osteonectin, α-tubulin, and GAPDH, and decreased expression of glutathione peroxidase mRNA in 3 anaplastic carcinomas (Figure 1).

### DISCUSSION

In this study, we used SAGE to analyse cDNAs from tissues of a normal thyroid and 4 thyroid tumours and created expression profiles for each tissue. In our results, some tag sequences corresponded to more than one gene. It was not possible, by means of only SAGE-data analysis, to determine whether all of the corresponding genes were expressed in the tissue. In the case of these sequences, further analyses, such as Northern blot or quantitative RT-PCR analyses, may be needed. Some tag sequences with no homology to known genes appear on the list. These sequences might be derived from some unknown genes, although the possibility of interference by the individual variations in the 3′ untranslated region of mRNAs should be also considered.

Pauws et al recently described the application of SAGE to create an expression profile of the normal thyroid (Pauws et al, 2000). Their data are quite similar to ours in that the majority of the highly expressed sequences coded mitochondorial or ribosomal proteins and the thyroglobulin gene was highly expressed. However, while they detected 24 tags of thyroid peroxidase, we detected none in the normal thyroid tissue and only 2 in the follicular adenoma. The effects of some endemic factors, such as iodine uptake, may explain this discrepancy. Further, because we performed SAGE analysis on a smaller scale than they did, only about 600 unique genes were identified. Thus, the analyses were limited to abundantly expressed sequences, and this is another reason why most of the thyroid-specific genes with moderate or low expression levels could not be detected.

In our study, the tag sequences of some genes, such as thyroglobulin, cathepsin B, and thymosin beta 10, were expressed in the benign and malignant tumours in a manner similar to that in previous reports (Brabant et al, 1991; Shuja and Murnane, 1996; Califano et al, 1998), suggesting the reliability of these SAGE data. For example, the tag sequence of cathepsin B occurred at a much higher rate in the papillary and follicular carcinomas than in the normal thyroid or the follicular adenoma.

In the anaplastic carcinomas, most of the highly occurring tag sequences code mitochondorial proteins, ribosomal proteins, or housekeeping genes, such as GAPDH. Interestingly, the products of some of these genes are already being used as serum tumour markers such as beta 2-microglobulin and ferritin. Thus, some of the genes identified here and shown to have high occurrence rates in thyroid carcinomas might be used as serum tumour markers of thyroid malignancies.

Osteonectin is a bone matrix protein synthesized by cells of the osteoblastic lineage, with a possible association having been suggested between this protein and microcalcifications in some malignant tissues (Bellahcene and Castronovo, 1995). The corresponding tag sequence of osteonectin mRNA showed a high occurrence rate in anaplastic carcinoma, and over-expression of this gene was confirmed by semi-quantitative RT-PCR. Osteonectin expression may become a new marker of anaplastic carcinomas, and the relationship between the expression of osteonectin and these cancers' aggressive biological characteristics may provide an interesting focus of study.

One of the most difficult distinctions in thyroid pathology is the differentiation between benign follicular adenomas and follicular carcinomas (Rosai and Carcangiu, 1987). Preoperative differentiation of follicular adenomas and carcinomas by cytopathological examination is quite difficult; accordingly, there has been a concentrated effort to establish a definite molecular marker of follicular carcinoma. Although only several differentially expressed genes were identified in the present study, some of the genes with known and unknown properties as listed in Table 3 may be candidate markers of follicular carcinomas.

In conclusion, in the present report, we analysed the gene expression profiles in the normal thyroid and 4 representative thyroid neoplasms. The results of this study may provide clues toward not only the establishment of a molecular-based diagnosis and therapy, but also an improved understanding of thyroid function and tumorigenesis.

### ACKNOWLEDGEMENT

This work was supported by a Grant-in-Aid for Encouragement of Young Scientists (to TT; No. 10771346) from the Ministry of Education, Science, Sports and Culture of Japan. We thank Hiromi Takarada and Ikuhiro Maeda for technical assistance and Dr Kenneth Kinzler for providing us the SAGE software.

### REFERENCES

Arcari P, Martinelli R and Salvatore F (1984) The complete sequence of a full length cDNA for human liver glyceraldehyde-3-phosphate dehydrogenase: evidence for multiple mRNA species. *Nucleic Acids Res* **12:** 9179–9189

Bellahcene A and Castronovo V (1995) Increased expression of osteonectin and osteopontin, two bone matrix proteins, in human breast cancer. *Am J Pathol* **146:** 95–100

Brabant G, Maenhaut C, Kohrle J, Scheumann G, Dralle H, Hoang-Vu C, Hesch RD, von zur Muhlen A, Vassart G and Dumont JE (1991) Human thyrotropin receptor gene: expression in thyroid tumors and correlation to markers of thyroid differentiation and dedifferentiation. *Mol Cell Endocrinol* **82:** R7–12

Califano D, Monaco C, Santelli G, Giuliano A, Veronese ML, Berlingieri MT, de Franciscis V, Berger N, Trapasso F, Santoro M, Viglietto G and Fusco A (1998) Thymosin beta-10 gene overexpression correlated with the highly malignant neoplastic phenotype of transformed thyroid cells in vivo and in vitro. *Cancer Res* **58:** 823–828

Chiappetta G, Tallini G, De Biasio MC, Manfioletti G, Martinez-Tello FJ, Pentimalli F, de Nigris F, Mastro A, Botti G, Fedele M, Berger N, Santoro M, Giancotti V and Fusco A (1998) Detection of high mobility group I HMGI(Y) protein in the diagnosis of thyroid tumors: HMGI(Y) expression represents a potential diagnostic indicator of carcinoma. *Cancer Res* **58:** 4193–4198

Chomczynski P and Sacchi N (1987) Single-step method of RNA isolation by acid guanidinium thiocyanate- phenol-chloroform extraction. *Anal Biochem* **162:** 156–159

Cowan NJ, Dobner PR, Fuchs EV and Cleveland DW (1983) Expression of human alpha-tubulin genes: interspecies conservation of 3′ untranslated regions. *Mol Cell Biol* **3:** 1738–1745

de Nigris F, Visconti R, Cerutti J, Califano D, Mineo A, Santoro M, Santelli G and Fusco A (1998) Overexpression of the HIP gene coding for a heparin/heparan sulfate-binding protein in human thyroid carcinomas. *Cancer Res* **58:** 4745–4751

Farid NR (1996) Molecular pathogenesis of thyroid cancer: the significance of oncogenes, tumor suppressor genes, and genomic instability. *Exp Clin Endocrinol Diabetes* **104:** 1–12

Gonsky R, Knauf JA, Elisei R, Wang JW, Su S and Fagin JA (1997) Identification of rapid turnover transcripts overexpressed in thyroid tumors and thyroid cancer cell lines: use of a targeted differential RNA display method to select for mRNA subsets. *Nucleic Acids Res* **25:** 3823–3831

Hedinger C, Williams ED and Sobin LH (1989) The WHO histological classification of thyroid tumors: a commentary on the second edition. *Cancer* **63:** 908–911

Hibi K, Liu Q, Beaudry GA, Madden SL, Westra WH, Wehage SL, Yang SC, Heitmiller RF, Bertelsen AH, Sidransky D and Jen J (1998) Serial analysis of gene expression in non-small cell lung cancer. *Cancer Res* **58:** 5690–5694

Matsubara K and Okubo K (1993) cDNA analyses in the human genome project. *Gene* **135:** 265–274

© 2000 Cancer Research Campaign

Pauws E, Moreno JC, Tijssen M, Baas F, de Vijlder JJ and Ris-Stalpers C (2000) Serial analysis of gene expression as a tool to assess the human thyroid expression profile and to identify novel thyroidal genes. *J Clin Endocrinol Metab* **85:** 1923–1927

Rosai J and Carcangiu ML (1987) Pitfalls in the diagnosis of thyroid neoplasms. *Pathol Res Pract* **182:** 169–179

Shuja S and Murnane MJ (1996) Marked increases in cathepsin B and L activities distinguish papillary carcinoma of the thyroid from normal thyroid or thyroid with non-neoplastic disease. *Int J Cancer* **66:** 420–426

Swaroop A, Hogan BL and Francke U (1988) Molecular analysis of the cDNA for human SPARC/osteonectin/BM-40: sequence, expression, and localization of the gene to chromosome 5q31-q33. *Genomics* **2:** 37–47

Takahashi K, Akasaka M, Yamamoto Y, Kobayashi C, Mizoguchi J and Koyama J (1990) Primary structure of human plasma glutathione peroxidase deduced from cDNA sequences. *J Biochem* **108:** 145–148

Takano T, Matsuzuka F, Sumizaki H, Kuma K and Amino N (1997) Rapid detection of specific messenger RNAs in thyroid carcinomas by reverse transcription-PCR with degenerate primers: specific expression of oncofetal fibronectin messenger RNA in papillary carcinoma. *Cancer Res* **57:** 3792–3797

Takano T, Miyauchi A, Yokozawa T, Matsuzuka F, Liu G, Higashiyama T, Morita S, Kuma K and Amino N (1998) Accurate and objective preoperative diagnosis of thyroid papillary carcinomas by reverse transcription-PCR detection of oncofetal fibronectin messenger RNA in fine-needle aspiration biopsies. *Cancer Res* **58:** 4913–4917

Takano T, Miyauchi A, Yokozawa T, Matsuzuka F, Maeda I, Kuma K and Amino N (1999) Preoperative diagnosis of thyroid papillary and anaplastic carcinomas by real-time quantitative reverse transcription-polymeras chain reaction of oncofetal fibronectin messenger RNA. *Cancer Res* **59:** 4542–4545

Velculescu VE, Zhang L, Vogelstein B and Kinzler KW (1995) Serial analysis of gene expression. *Science* **270:** 484–487

Zhang L, Zhou W, Velculescu VE, Kern SE, Hruban RH, Hamilton SR, Vogelstein B and Kinzler KW (1997) Gene expression profiles in normal and cancer cells. *Science* **276:** 1268–1272

 © 2000 Cancer Research Campaign
